# Disrupting the status quo to achieve early inclusion of pregnant women in studies of new agents for prevention and treatment of HIV infection

**DOI:** 10.1002/jia2.25927

**Published:** 2022-07-19

**Authors:** Elaine J. Abrams, Martina Penazzato

**Affiliations:** ^1^ ICAP at Columbia University Mailman School of Public Health Columbia University New York New York USA; ^2^ Vagelos College of Physicians and Surgeons Columbia University New York City New York USA; ^3^ Global Programme on HIV, Hepatitis and Sexually Transmitted infections World Health Organization Geneva Switzerland

**Keywords:** pregnant women, antiretroviral treatment, human immunodeficiency virus, HIV prevention, pharmacokinetics, breastfeeding women

In 2021, long‐acting cabotegravir (LA CAB) was approved by the United States Food and Drug Administration for an indication of pre‐exposure prophylaxis in women [1]. This is a first in what is hoped to be a robust pipeline of long‐acting antiretroviral agents heralding a new era in HIV prevention [2]. The highly anticipated rollout of this agent among populations of young women with persistently high HIV incidence and fertility rates can be expected to leapfrog efforts to end the AIDS epidemic, protecting young women from new HIV infections and reducing new paediatric infections, which are increasingly attributed to incident maternal infections during pregnancy and breastfeeding [3].

The introduction of LA CAB presents the global HIV community with a significant challenge. To date, there are only sparse data on use of this agent during pregnancy and breastfeeding. Women enrolled in the seminal trial, HPTN 084, were required to use contraception and the few women who became pregnant discontinued the study agent [4]. There are some pharmacokinetic (PK) and safety data from the 26 women who became pregnant on LA CAB, but minimal information on pregnancy or birth outcomes, or on repeated dosing during pregnancy and breastfeeding [5]. Pregnancy PK studies are now underway as part of the open label extension of HPTN 084, which has dropped the contraception requirement and will allow women who become pregnant to continue LA CAB. Nonetheless, these delays leave the global health community in a quandary: we are anxious to introduce this transformative product to protect highly vulnerable people from new HIV acquisitions, but we have inadequate information on use of this drug during pregnancy. What can be said to the healthcare providers dispensing this agent, and to the young women considering its use about the risks and benefits to her health and the health of her baby should she become pregnant? Should she be advised to defer pregnancy until more is known about this agent? If she is considering pregnancy, should she also consider an alternative agent for HIV prevention? What effects, short‐ and long‐term, can be expected for the child if their mother is on LA CAB during pregnancy?

Neither the investigators nor the pharmaceutical companies can be faulted for this unfortunate situation. They applied the highest ethical standards and followed current regulatory guidance aimed at protecting the foetus, at all costs, from potentially harmful exposures during pre‐licensure studies [6]. The situation is also not unique to this agent, but is another example of the consequences of long delays in studying new agents in pregnancy [7]. The same situation has repeated itself again and again for new products, most recently for vaccines and other agents for prevention and treatment of SARS‐CoV‐2 infections [8].

Ongoing debate in the scientific community is clearly emphasizing that excluding pregnant people from participation in pre‐licensure studies achieves the goal of protecting the foetus from harm, but only during the trial period. The undefined risk persists once the drug is approved, and it is simply transferred from the more controlled environment of a trial setting to routine care, where the mother and prescribing healthcare worker are left to make decisions with little if any information. This practice does not just challenge the clinical management and the need to address the immediate health needs of the mother, but substantially affects the capacity to observe and measure safety for the mother and child, which is, at best, significantly delayed and deeply compromised [7].

There is an emerging consensus that this historical approach must be reconsidered and revised. Multiple investigators, clinicians and policy makers have pointed out the stark inequities and delineated the clinical consequences of the current approach [9, 10, 11]. The United States Task Force on Research Specific to Pregnant Women and Lactating Women has identified the need to increase the quantity, quality and timeliness of research on therapeutic products used by pregnant women and reduce regulatory barriers and liability to facilitate an evidence base for new therapeutic products that may be used by women who are or may become pregnant and by lactating women [12]. The Pregnancy and HIV/AIDS: Seeking Equitable Study developed ethical guidance for advancing responsible HIV/co‐infection research with pregnant women [13]. Importantly, they proposed to reframe the current approach from (1) pregnant women as a vulnerable population to a complex population; (2) protection from research to protection through research; and (3) presumptive exclusion to fair inclusion. This framework should guide a transformation of the current paradigm around inclusion of pregnant women in HIV research.

But how does one attain the changes needed to accelerate the inclusion of pregnant women in trials of new antiretrovirals? What are the core principles and the tangible, actionable steps towards real equity in research and, ultimately, equity in access to new HIV medications for this population? We have spent the last year convening stakeholders in a collaborative, iterative process discussing the many complex issues and critical next steps to take theory to action. This supplement gathers the key considerations made by examining this approach and exploring how to rapidly change the status quo. Penazzato et al., in this issue, describe this technical consultation, its high‐level outcomes as well as a set of concrete strategic actions intended to disrupt the status quo and accelerate the inclusion of pregnant women in studies of new agents for prevention and treatment of HIV infection [14].

The first critical step forward is to engage with the totality of risk, risks to the foetus and risks to the mother. Each new drug confers potential harm to the foetus as well as the child, with risk varying by timing and duration of exposure during pregnancy. Exposure at the time of conception and early gestation are associated with the greatest concerns of teratogenicity and miscarriage, while later and prolonged exposure may impact foetal growth and development. Furthermore, risk is not contained to the gestational period but may manifest across the life course due to direct or indirect effects of drug exposure [15, 16, 17]. At the same time, there are risks to the pregnant person, risks of pregnancy‐specific toxicities and importantly, risks of using the wrong dose if PK and dosing are not studied during pregnancy.

These risks, however, are no different during a trial versus the post‐trial period, but rather are part and parcel of introducing new medications. These risks can affect the entirety of the population should the lack of data prevent effective scale‐up of the product. It is, therefore, essential to fully engage with and address the nuances and complexities of risk rather than pushing them off until the post‐marketing period when risks, as well as outcomes, are less easily discerned. It is also of paramount importance to consider the spectrum of risks against the potential benefits that innovative treatment and preventive options can bring to the mother, her child and the community. These benefits depend on the characteristics of the new medication as well as the existing available options. New agents can bring considerable advantages for preventing HIV incident infection or controlling HIV viremia, and in turn preventing vertical transmission and improving maternal outcomes. A careful assessment of the risk and benefit balance is, therefore, essential when approaching the issue of investigating new medications in people of reproductive age. Singh et al., in the supplement, apply an ethical lens to these issues and address the question of maternal autonomy in this balancing act [18].

On this basis, what data are needed to safely use a product in pregnancy? The required non‐clinical studies must be completed and should demonstrate no relevant negative effects. Non‐clinical studies, however, are complex to understand and interpret, and questions persist about the translation of various animal models to humans. Based on early developmental and reproductive toxicology (DART) findings in tandem with several case reports, the antiretroviral efavirenz was withheld from millions of women of childbearing potential because of a potential link to neural tube defects [19, 20, 21]. Instead, nevirapine was the recommended anchor antiretroviral, an agent later determined to be associated with significantly worse pregnancy and birth outcomes compared with efavirenz [22]. After more than a decade from when the first findings were reported and with an adequate number of observations of preconception exposures, efavirenz was determined not to be associated with a higher risk of neural tube defect [23]. More work needs to be done to better interpret and apply findings from the non‐clinical studies. Greupink et al. discuss the optimal timing for these studies, their complex interpretation and the need to improve communication of the results and their implications [24].

The proper dose in pregnancy for all new agents should be determined before the drug is marketed. It should be known if any dosing adjustments are required and if there are any unexpected toxicities associated with the pregnant state. As described by Abrams et al. in this supplement, all new anti‐HIV agents intended for a prevention or treatment indication for adolescents and adults should be studied in pregnant populations for dosing and preliminary safety, with data available at the time of registration [25]. This can and should be achieved particularly for all high‐priority products with expected broad use. Knowing the proper dose would reduce some of the uncertainty women experience when they become pregnant on a new agent. The WHO is leading real‐time processes to regularly assess priority products for which accelerated study is warranted [26]. These same agents should also be prioritized for early pregnancy‐specific safety studies that assess adverse pregnancy and birth outcomes.

Innovation will be key to achieving greater equity and more timely inclusion of pregnant women in pre‐licensure studies. Greupink et al. propose alternative combinations of DART study designs and physiologically based PK modelling as complementary approaches to determine drug dosing and safety in pregnancy [24]. Similarly, Brummel et al. provide a compelling rationale for the importance of randomized clinical trials (RCTs) to study safety in pregnancy for all high‐priority agents, specifically safety outcomes uniquely important to the mother and infant [27]. The authors propose several innovative approaches to obtain pregnancy data in a timelier manner, such as using a composite study endpoint of adverse birth outcomes (prematurity, small for gestational age and foetal loss) and consideration of alternative RCT designs, such as adaptive clinical trials. Innovation in regulatory frameworks is another important enabler brought forward by Saint‐Raymond and Mofenson, who discuss the opportunities and limitations of existing frameworks and draw from the evolution of the paediatric regulation in Europe and the United States to recommend the establishment of a maternal investigation plan that formalizes systematic collection of pregnancy data as part of the clinical development plan of any new chemical entity seeking market authorization [28].

As the research community moves towards a more proactive approach to investigation of new HIV medications in pregnancy, strengthening the surveillance safety net will continue to be a critical pillar to safe implementation, acknowledging that the full safety profile of an agent used in pregnancy may take thousands of exposures to determine lack of harm [29]. We, the scientific community, regulatory, pharmaceuticals and the community, will need to shoulder this uncertainty. We can only do this if we can rely on an enhanced safety net to identify these potential problems over time with purposeful data collection and harmonized tools, and coordinate analyses that maximize quality and use of post‐marketing data as described in Renaud et al. [29].

None of this, however, will become a reality without the guidance and involvement of women and mothers. Their perspective will not just contribute to the design of patient‐centred solutions, but will be a powerful tool to design trials, support women who may wish to participate in those studies and most importantly to make sure that results are well articulated and promptly shared with the community of people living with or at risk of acquiring HIV. Clayden et al. put the spotlight on the voice of women and mothers and outline a number of approaches to enable a meaningful engagement across the various stages of design, implementation, analysis and dissemination of the research that relates to the use of any new medications for treatment and or prevention of HIV [30].

Now, more than ever–at a time when the global health community is confronted with the excitement of rapid medical innovation as well as the challenges and responsibilities of ensuring equitable access to all in need–we must take action together to transform the current paradigm and ensure that no women and mothers are left behind [31]. We must fast‐forward to a future when women of reproductive age will no longer shoulder the burden of having fewer options to stay HIV‐free or remain healthy and break the chain of HIV transmission. This supplement charts a path forward from theory to action—to reach equity and to end the HIV epidemic.

## COMPETING INTERESTS

No competing interests to report.

## AUTHORS’ CONTRIBUTIONS

EJA and MP developed the concept for this editorial, drafted and finalized the manuscript.

## DISCLAIMER

The views expressed in this article are the personal views of the author(s) and may not be understood or quoted as being made on behalf of or reflecting the position of the regulatory agency/agencies or organizations with which the author(s) is/are employed/affiliated.
